# Distribution of cionin, a cholecystokinin/gastrin family peptide, and its receptor in the central nervous system of *Ciona intestinalis* type A

**DOI:** 10.1038/s41598-024-55908-7

**Published:** 2024-03-15

**Authors:** Shiho Taniguchi, Satoshi Nakayama, Rin Iguchi, Yasunori Sasakura, Honoo Satake, Shuichi Wada, Nobuo Suzuki, Michio Ogasawara, Toshio Sekiguchi

**Affiliations:** 1 Noto Marine Laboratory, Institute of Nature and Environmental Technology, Kanazawa University, Ogi, Noto-Cho, Ishikawa 927-0553 Japan; 2 Department of Biology, Graduate School of Science, Chiba University, 1-33 Yayoi-Cho, Inage-Ku, Chiba, 263-8522 Japan; 3 Shimoda Marine Research Center, University of Tsukuba, 5-10-1 Shimoda, Shizuoka, 415-0025 Japan; 4 Bioorganic Research Institute, Suntory Foundation for Life Sciences, Seikacho, Kyoto, 619-0284 Japan; 5 Department of Animal Bioscience, Faculty of Bioscience, Nagahama Institute of Bio-Science and Technology, Nagahama, Shiga 526-0829 Japan

**Keywords:** Cholecystokinin, Gastrin, Ascidian, Cholinergic neuron, Neurophysiology, Peptide hormones

## Abstract

The cholecystokinin (CCK)/gastrin family peptides are involved in regulation of feeding and digestion in vertebrates. In the ascidian *Ciona intestinalis* type A (*Ciona robusta*), cionin, a CCK/gastrin family peptide, has been identified. Cionin is expressed exclusively in the central nervous system (CNS). In contrast, cionin receptor expression has been detected in the CNS, digestive tract, and ovary. Although cionin has been reported to be involved in ovulation, its physiological function in the CNS remains to be investigated. To elucidate its neural function, in the present study, we analyzed the expression of cionin and cionin receptors in the CNS. Cionin was expressed mainly in neurons residing in the anterior region of the cerebral ganglion. In contrast, the gene expressin of the cionin receptor gene *CioR1*, was detected in the middle part of the cerebral ganglion and showed a similar expression pattern to that of *VACHT*, a cholinergic neuron marker gene. Moreover, *CioR1* was found to be expressed in cholinergic neurons. Consequently, these results suggest that cionin interacts with cholinergic neurons as a neurotransmitter or neuromodulator via CioR1. This study provides insights into a biological role of a CCK/gastrin family peptide in the CNS of ascidians.

## Introduction

In mammals, cholecystokinin (CCK) and gastrin are peptides that share common molecular features such as the sulfated tyrosine residue and the common amidated tetrapeptide Trp-Met-Asp-Phe-NH_2_ in the C-terminus^1,2^. Mammalian CCK, released from the duodenum, is involved in gallbladder contraction^1,3^. CCK also functions as a neuropeptide in the brain. For instance, CCK is associated with the enhancement of emotional and memory behaviors^4^ and reduction in food intake^4–6^. In addition, gastrin is secreted in the stomach and stimulates gastric acid release in mammals^7,8^. Thus, CCK and gastrin have differential functional roles, although they share common molecular features conserved in vertebrates. Furthermore, synteny analysis using vertebrate genome information revealed that *CCK* and *gastrin* loci are expected to originate from a single chromosomal locus and be generated by genome duplication^9^. CCK/gastrin family peptide excert their effects in mammals via two paralogous receptors known as CCK1 receptor (CCK1R) and CCK2 receptor (CCK2R)^10,11^. CCK binds to both CCK1R and CCK2R, whereas gastrin binds specifically to CCK2R^11^. These two types of receptors are widely conserved in vertebrates, as is the case for ligand peptides^12^. Collectively, CCK/gastrin family peptides and their receptors are conserved in vertebrates.

CCK/gastrin family peptides are also widely conserved in bilaterians such as Arthropoda, Nematoda, Mollusca, Hemichordata, Echinodermata, and Chordata^13,14^. For example, sulfakinin (SK) and NLP-12 were identified in insects and nematodes, respectively ^15,16^. These peptides contain a sulfated tyrosine residue and activate CCKR homologous receptors. However, the C-terminal tetrapeptide sequences of SK (His-Met-Arg-Phe-NH_2_) and NLP-12 (Pro-Leu-Gln-Phe-NH_2_) are slightly different from that of vertebrate CCK/gastrin (Trp-Met-Asp-Phe-NH_2_). Therefore, SK and NLP-12 are considered to be protostome-type CCK/gastrin family peptides.

Cionin is an 8-amino acid peptide purified from the neural complex, the central nervous system (CNS) of *C. intestinalis* type A^17^, and has two sulfated tyrosine residues at the 2nd and 3rd positions from the N-terminal region and the C-terminal sequence (Trp-Met-Asp-Phe-NH_2_), which is conserved in vertebrates^17^.

To clarify the origin and evolution of the vertebrate CCK/gastrin family, we have investigated cionin. In a previous study, we identified two cionin receptors, *CioR1* and *2*, in *Ciona*. CioR1 and 2 were shown to equipotently trigger intracellular calcium mobilization in response to cionin^18^. Moreover, our molecular phylogenetic analysis demonstrated that *CioR1* and *2* were derived from a common ancestor of chordate CCKRs and diverged in the *Ciona*-specific lineage^18^. These findings indicate that *cionin* and *CioRs* are orthologs of the vertebrate CCK/gastrin family peptides and their receptors, respectively, and that vertebrate-type CCK/gastrin family peptide emerged from ancestral chordates, not from ancestral vertebrate. In the previous study, we also verified that cionin was expressed mainly in the cerebral ganglion of the neural complex. However, the precise distribution of cioninergic neurons and their physiological functions in the central nervous system remain unclear.

In the present study, we elucidated the localization of cionin peptide and *CioR *mRNAs in the neural complex and the neural interaction between cionergic neurons and cholinergic neurons. These results are expected to provide crucial clues to the investigation of the biological roles of cionin in the central nervous system of ascidians.

## Results

### Distribution of cioninergic neurons in the neural complex

We previously performed expression analysis of *cionin* mRNA and peptide in the *Ciona* adult neural complex^18^. The neural complex comprises the cerebral ganglion and neural gland^19^. The cerebral ganglion is the CNS of ascidians, whereas the neural gland is a non-neuronal organ located on the ventral side of the cerebral ganglion ^20^. The previous study showed that cionin is expressed in the cerebral ganglion. However, the precise localization of cionin in the cerebral ganglion remains to be elucidated.

**Figure 1:**
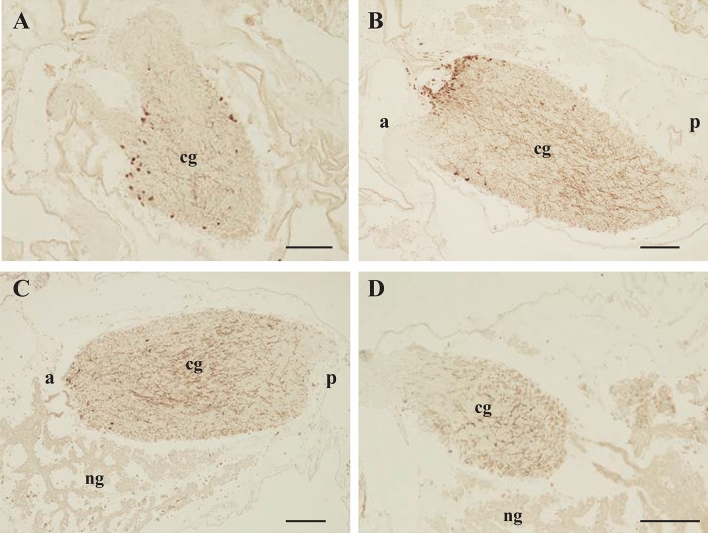
Immunohistochemical analysis of cionin in serial sections of the *Ciona* neural complex. (**A**) Section containing the anterior part of the neural complex, (**B**) Section containing the anterior and central parts of the neural complex, (**C**) Section containing the central and posterior parts of the neural complex, (**D**) Section containing the posterior part of the neural complex. The cg and ng show cerebral ganglion and neural gland, respectively. The a and p describe anterior and posterior, respectively. Scale bars represent 100 µm.

In the present study, we evaluated the precise distribution of cionin peptides in the neural complex by immunohistochemical analysis of serial sections of the neural complex using anti-cionin antibody. In sections that included the anterior region, cionin-immunopositive cell bodies were accumulated in the anterior part of the cerebral ganglion (Figs. 1A,B). Moreover, cionin-positive fibers were observed in the middle and posterior parts of the cerebral ganglion (Figs. 1B–D). The distribution of cionin-immunopositive neurites was detected mainly in the inner layer of the cerebral ganglion, whereas a part of cionin immunopositive nerve fivers projected toward the outer layer of the cerebral ganglion (Figs. 1B,C). These results revealed that the cioninergic neurons possess a cell body in the anterior region, and their fibers are distributed in the middle and posterior regions of the cerebral ganglion.

### Localization of *CioR1* and *2* mRNA in juvenile and adult neural complex

In our previous study, *CioR1* mRNA was shown to be expressed in the neural complex, ovary, stomach, intestine, oral siphon, and atrial siphon, and *CioR2* mRNA was detected in the neural complex, endostyle, ovary, oral siphon, and atrial siphon^18^.

**Figure 2:**
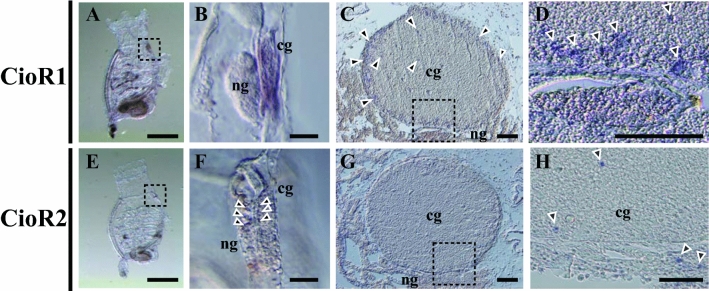
Localization of *CioR1* mRNA analyzed by in situ hybridization analysis. WISH of *CioR1* mRNA was performed in the juvenile (**A**) and adult neural complexes (**C**). Magnified view of black dotted boxes in A and C is presented in B and D, respectively. WISH of *CioR2* mRNA in the juvenile and adult neural complex show in E and G, respectively. Magnified photo of black dotted boxes in E and G is depicted in F and H, respectively. Arrowheads show positiv signals. cg, cerebral ganglion; ng, neural gland. Scale bars in A and E are 500 µm. Scale bars in C and G are 200 µm, whereas scale bars in B, D, F, and H are 100 µm.

To evaluate the localization of *CioR1* and *2* mRNA in the neural complex, we performed whole mount in situ hybridization (WISH) on juvenile *Ciona*. *CioR1* mRNA was predominantly localized to the cerebral ganglion (Figs. 2A,B). In addition, WISH of the adult neural complex demonstrated that the *CioR1* mRNA was detected in the outer layer of the cerebral ganglion, which mainly consists of the cell bodies of neurons (Figs. 2D,E). On the other hand, the signal of *CioR2* mRNA was detected in a limited number of cerebral ganglion cells and the neural gland (Figs. 2F,G). This localization pattern was also detected in the adult neural complex (Figs. 2H,I). These findings clarified that *CioR1 *and *2* are expressed dominantly in the cerebral ganglion and neural gland, respectively.

### Evaluation of the co-expression of *cionin*, *CioR1*, and *VACHT*

**Figure 3:**
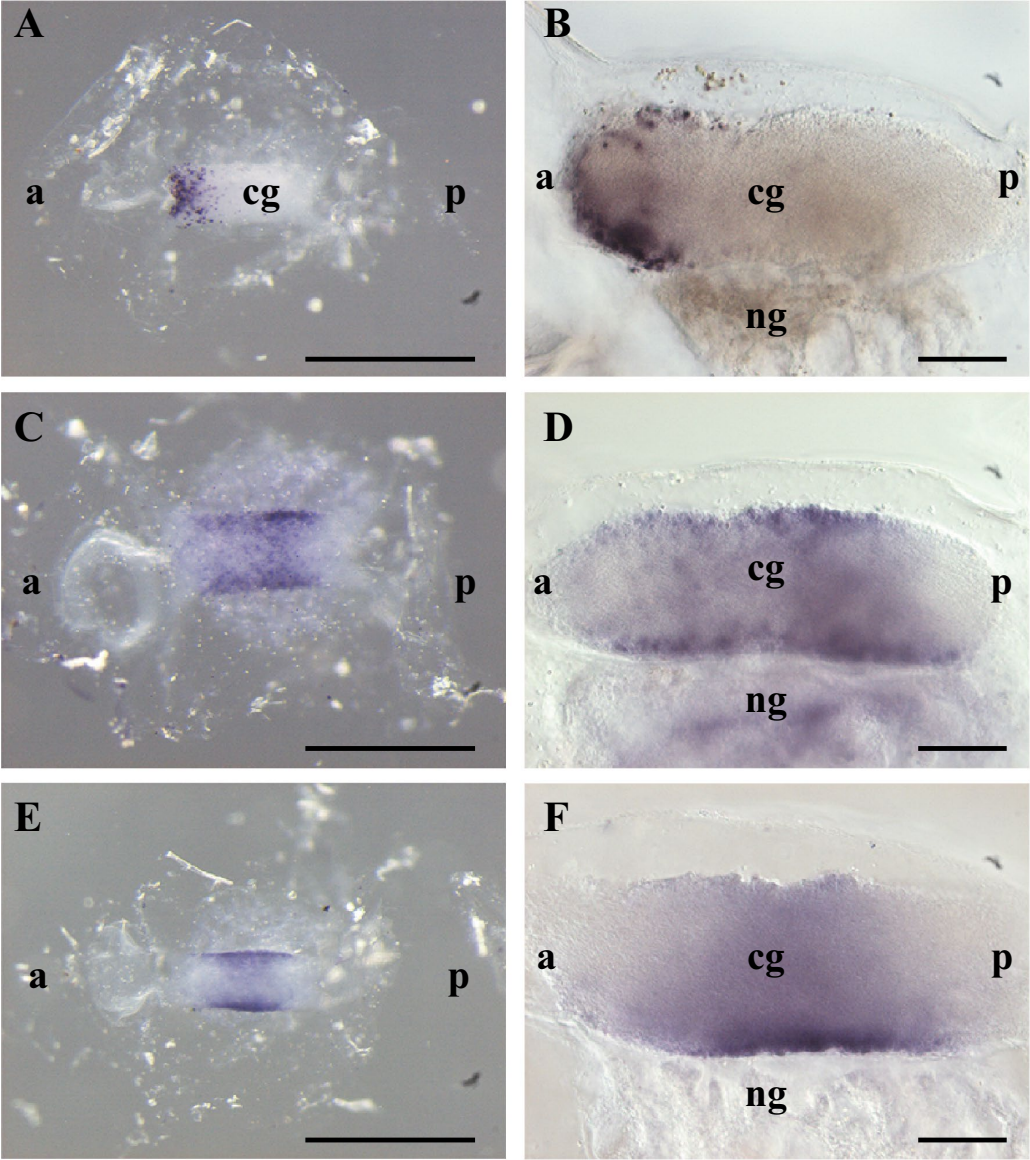
Localization analysis of *cionin*, *CioR1*, and *VACHT* in the adult neural complex. WISH of *cionin*, *CioR*1, and *VACHT* mRNA in the adult neural complex is shown in A, C, and E, respectively. B, D, and F represent sections A, C, and E, respectively. Scale bars in A, C, and E and B, D, and F are 1000 and 200 µm, respectively.

To confirm the cionin and CioR1-expressing regions, we performed WISH of the adult neural complex. *Cionin* mRNA expression was observed in the anterior region of the cerebral ganglion (Figs. 3A,B). The *CioR1* mRNA was expressed in the central region of the cerebral ganglion (Figs. 3C,D). The spatial expression pattern of *CioR1* mRNA was similar to that of *VACHT* mRNA, a marker of cholinergic neurons (Figs. 3E,F).

**Figure 4:**
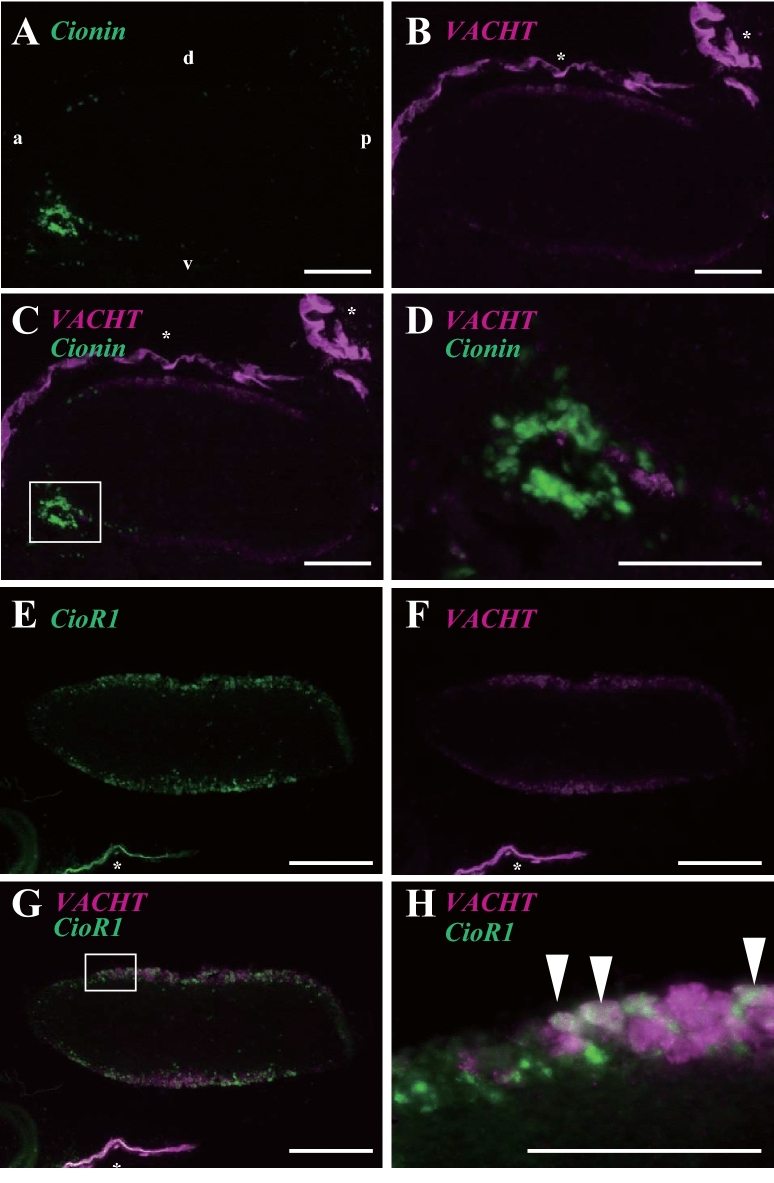
Double in situ hybridization analysis of *cionin*, *CioR1*, and *VACHT* mRNA in the adult neural complex. In situ hybridization of *cionin* (**A**) and *VACHT* (**B**). C shows double staining for *cionin* and *VACHT* mRNA. D shows an enlarged view of the white box in C. In situ hybridization of *CioR1* (**E**) and *VACHT* (**F**). G represents double in situ hybridization of *CioR1* and *VACHT* mRNA. The enlarged view of the white box in G is depicted in H. Cells expressing both *CioR1* and *VACHT* were shown by white arrowheads. Nonspecific staining in the epithelia is indicated by an asterisk. The cg and ng show cerebral ganglion and neural gland, respectively. The a and p describe anterior and posterior, respectively. The d and v depict dorsal and ventral, respectively. Scale bars in A, B, C, E, F, and G represent 200 µm. Scale bars in D and H are 100 µm.

To clarify the co-expression of *cionin* and *CioR1* mRNA with *VACHT* mRNA, we conducted double in situ hybridization of the neural complex. Double in situ hybridization using *cionin* and *VACHT* probes indicated that *cionin*-positive signals did not overlap with *VACHT* signals in the cerebral ganglion as the result of WISH analysis (Figs. 4A–D). Next, we performed double in situ hybridization analysis using *CioR1* and *VACHT* probes. In the middle of the cerebral ganglion, cells co-expressing *CioR1* and *VACHT* were detected, since the *CioR1* mRNA was detected in *VACHT*-positive cell population (Figs. 4E–I). These results demonstrated that *CioR1* is expressed in several cholinergic neurons.

## Discussion

Ascidians are the extant animal group closest to vertebrates^21^. In addition to its phylogenetic position, genome information of some ascidians has been available in the public database^22,23^. In particular, *C. intestinalis* type A is a useful model for ascidians. Furthermore, various developmental engineering technologies, such as transgenic and genome-editing techniques, have been established in *Ciona*^22,24^. Therefore, *Ciona* is considered to be a primitive chordate model in both evolutionary and developmental biology. Recently, studies on the evolution of the nervous and endocrine systems have made progress in *Ciona*. Peptides and their receptors, which are prerequisites for the regulation of the nervous and endocrine systems, have been identified ^25–27^. Furthermore, multiple *Ciona*-specific peptide-receptor pairs have thus far been elucidated^28^. These findings indicate that *Ciona* also has been regarded as a model animal to study the evolution of the endocrine and nervous systems in chordates. We have investigated *Ciona* CCK/gastrin family peptide, cionin. Since cionin has vertebrate-type CCK/gastrin family peptide characteristics in its primary structure, the investigation of the neural function of cionin will contribute to clarifying the origin and diversification of CCK/gastrin family peptides in vertebrates. Our previous study uncovered the tissue distribution of *cionin*, *CioRs* mRNA, and the ligand-receptor relationship between cionin and CioRs^18^. Recently, the peripheral action of cionin has been elucidated. Osugi et al. showed that cionin elicits ovulation via CioR2 expressed in *the Ciona* ovarian follicle cells^29^. However, a biological function of cionin in the central nervous system remains unsolved. Therefore, we analyzed the localization of cionin and CioRs in the central nervous system in detail.

Cionin-immunopositive neurons and nerve fibers were detected in the anterior and middle/posterior regions of the cerebral ganglion, respectively, suggesting that these fibers run from the cell body in the anterior part of the cerebral ganglion. WISH analysis of *CioRs* revealed that *CioR1* mRNA was localized throughout the entire cerebral ganglion of juvenile and adult neural complex (Figs. 2A–D). In contrast, transcripts of *CioR2* were detected mainly in the neural gland of juvenile and adult neural complex (Figs. 2E–G). Since the expression of *CioR2* was non-neuronal, CioR2 was excluded from the subject of further research. The expression of *cionin* mRNA in the neural complex was compared with that of *CioR1* mRNA by WISH (Fig. 3). *Cionin* mRNA expression was restricted to the anterior part of the cerebral ganglion in accordance with the result of the immunohistochemistry of the serial section of the neural complex (Figs. 1B, 3A,B). *CioR1* mRNA localization was observed in the central part of the cerebral ganglion. The spatial expression pattern of *CioR1* mRNA was similar to that of the cholinergic neuron marker, *VACHT* mRNA (Figs. 3C–F). Double in situ hybridization demonstrated that several the cholinergic neuronal cell bodies expressed CioR1 in the central region (Fig. 4). Additionally, cionin-immunopositive fibers were detected in the vicinity of the outer layer of the middle part of the cerebral ganglion, where CioR1-positive cholinergic neurons were observed. In mammals, CCK is associated with cholinergic neurons or acetylcholine-expressing cells as a transmitter or modulator^30,31^. For instance, CCK-8 modulates the release of acetylcholine in the rat striatum^30^. These findings indicate the possibility that cioninergic neurons regulate the function of cholinergic neurons via CioR1. Further studies on the direct interaction between CioR1-expressing cholinergic and cioninergic neurons are necessary.

Cholinergic neuron distribution has been reported using the *VACHT* promoter-driven Kaede marker transgenic juvenile *Ciona*^32^ and have been detected throughout the juvenile cerebral ganglion^32^. These findings seem to be in contrast with the present WISH analysis (Fig. 4). However, the major expression area of the *VACHT* promoter-driven Kaede was observed in the central region, similar to the expression area of *VACHT* detected by WISH (Fig. 4). It has been reported that acetylcholine released from the cholinergic nerve terminals is involved in the contraction of ascidian body muscle^33,34^. Moreover, expression analysis using the *Ciona* marker transgenic line driven by the *VACHT* promoter demonstrated that cholinergic neurons originating from the middle region project to the body wall muscle, gills, oral siphon, and atrial siphon at the juvenile stage^32^. Optogenetic analysis using the channel rhodopsin expressed under the *VACHT* promoter uncovered that cholinergic neurons derived from the middle part of the ganglion act as motor neurons and are essential for the contraction of the body wall muscle, oral/atrial siphon, and ciliary movement of the gill slit^32^. Furthermore, a pharmacological study using juvenile *Ciona* demonstrated that acetylcholine is involved in the arrest of the gill cilia^35^. These findings suggest that cholinergic neurons expressing CioR1 also act as motor neurons, and cionin regulates motor neurons through CioR1. In addition, cholinergic neurons also play a pivotal role in the various region of the brain^13^. However, these functions have yet to be elucidated in ascidians. Therefore, distribution and physiological function of CioR1-expressing cholinergic neurons in *Ciona* await further investigation.

In summary, we clarified the localization of cioninergic neurons in the cerebral ganglion and the presence of CioR1-expressing cholinergic neurons. This study paves the way for investigating the role of neural peptides in the *Ciona* central nervous system.

## Materials and methods

### Animals

*Ciona intestinalis* type A (*Ciona robusta*) was purchased from the National Bio-Resource Project (NBRP) *Ciona*, which is provided from the Maizuru Fisheries Research Station of Kyoto University or Misaki Marine Biological Station of the University of Tokyo. The ascidians were maintained in the aquarium of the Noto Marine Laboratory.

### Whole mount in situ hybridization of *Ciona* juvenile and adult neural complex

WISH analysis of *Ciona* juvenile and adult neural complexes was performed as described previously^36^. DIG-labeled RNA probes for *cionin* and *CioR1* were synthesized using a DIG RNA-labeling kit (Roche, Basel, Switzerland). Primer sets for *cionin*, *CioR1*, *CioR2*, and *vesicular acetylcholine transporter* (*VACHT*)^37^ were designed using NM_001032539, AJ549433, NM_001278980 and KY21.Chr1.783.v1.SL1-1, respectively. The primer sets used for probe synthesis are listed in Table 1. WISH performed by using the sense probes did not show any expression signals (data not shown).

**Table 1:** Primer sequences.

Primer name (position)	Sequence
Cionin FW (15–34)	5′-TCGGTGATTGGTTTTTCTGG-3′
Cionin-RV (460–479)	5′-AGCATTGAGCGAAGAAAACG-3′
CioR1-FW (1179–1198)	5′-AAGTAACAGAAAGAGATGCG-3′
CioR1-RV (1741–1760)	5′-CGTGTACAACTAGCAACTGT-3′
CioR2-FW (1031–1050)	5′-CAGCGAATGCAACACTTCAC-3′
CioR2-RV (1571–1590)	5′-TAATCTGGCAAGGAGCATAG-3′
VACHT-FW (1301–1320)	5′-GTGTCTCTACCGCTGCTGTC-3′
VACHT-RV (2179–2200)	5′-GCCCAACTATTTTGGCACATGG-3′

### Immunohistochemistry of the neural complex in adult *Ciona*

Immunohistochemistry of the adult neural complex was performed as the previous study^18^. Briefly, the neural complex was obtained from an adult individual and fixed overnight in Bouin’s solution at room temperature. Ten µm paraffin sections were generated by microtome. After deparaffinization, the sections were incubated with a blocking solution containing 1% normal horse serum and 0.4% Triton X-100 in PBS at room temperature for 1 h. Anti-cionin rabbit antibody (500-fold dilution) in blocking solution was incubated at 4C overnight. The specificity of the antibody was already validated by antibody absorption test^18^. After rinsing with PBS, biotin-conjugated donkey anti-rabbit IgG (1000-fold dilution) was added to the sections and incubated at room temperature for 1 h. After the sections were rinsed with PBS, they were incubated with an avidin-biotinylated HRP complex (ABC, Vector Laboratories, Inc., Burlingame, CA) for 30 min. For color development, the sections were incubated with 0.02% 3,3-diaminobenzidine-tetrachloride and 0.06% hydrogen peroxide in 0.05 M Tris–HCl, pH 7.6) for several minutes. Finally, signals were observed by a BZ-9000 microscope (Keyence, Osaka, Japan).

### Double in situ hybridization analysis

Neural complexes were surgically removed from the juvenile specimens fixed in 4% paraformaldehyde. The sections were then dehydrated with ethanol and embedded in polyester wax (BDH; Poole, UK). Embedded neural complexes were sectioned at a thickness of 10 μm. Antisense *VACHT* RNA probes were synthesized using T7-RNA polymerase (Thermo Fisher Scientific, Pittsburgh, PA, USA) with DIG RNA Labeling Mix (Roche). Fluorescein RNA Labeled Mix (Roche) was used to synthesize antisense *cionin* and *CioR1* probes using T7-RNA polymerase. The primer sets used for probe synthesis and probe lengths are listed in Table 1. The wax from the neural complex sections was removed using an ethanol series, and the sections were used for in situ hybridization. Two-color in situ hybridization was performed as previously described^38^. First-color staining was performed using the TSA Plus Fluorescein System (PerkinElmer, Waltham, MA, USA) after incubation with anti-fluorescein-HRP (Perkin Elmer). Second-color staining was performed by TSA Plus Cyanine 3 System (PerkinElmer) after incubation with anti-DIG–POD (Roche). The expression signals were observed using an Olympus BX51 microscope (Olympus, Tokyo, Japan).

## Data Availability

The corresponding author will provide the data reported in the study if a request is reasonable. Sequence data of CioR2 previously was deposited in the GenBank with accession number AB669183.1

## References

[1] Rehfeld JF (2021). Cholecystokinin and the hormone concept. Endocr. Connect..

[2] Sekiguchi, T. in *Handbook of Hormones (Second Edition)* (eds Hironori Ando, Kazuyoshi Ukena, & Shinji Nagata) 301–303 (Academic Press, 2021).

[3] Sekiguchi, T. in *Handbook of Hormones (Second Edition)* (eds Hironori Ando, Kazuyoshi Ukena, & Shinji Nagata) 309–311 (Academic Press, 2021).

[4] Chen X (2019). Cholecystokinin release triggered by NMDA receptors produces LTP and sound–sound associative memory. Proc. Natl. Acad. Sci..

[5] D'Agostino G (2016). Appetite controlled by a cholecystokinin nucleus of the solitary tract to hypothalamus neurocircuit. Elife.

[6] Woods SC, May-Zhang AA, Begg DP (2018). How and why do gastrointestinal peptides influence food intake?. Physiol. Behav..

[7] Dockray G, Dimaline R, Varro A (2005). Gastrin: old hormone, new functions. Pflugers Arch..

[8] Sekiguchi, T. in *Handbook of Hormones (Second Edition)* (eds Hironori Ando, Kazuyoshi Ukena, & Shinji Nagata) 305–307 (Academic Press, 2021).

[9] Dupré D, Tostivint H (2014). Evolution of the gastrin-cholecystokinin gene family revealed by synteny analysis. Gen. Comp. Endocrinol..

[10] Dufresne M, Seva C, Fourmy D (2006). Cholecystokinin and gastrin receptors. Physiol. Rev..

[11] Zeng, Q., Ou, L., Wang, W. & Guo, D. Y. Gastrin, Cholecystokinin, Signaling, and Biological Activities in Cellular Processes. *Front. Endocrinol. (Lausanne)* **11**, 112, 10.3389/fendo.2020.00112 (2020). 10.3389/fendo.2020.00112 PMC7067705 32210918

[12] Yu N, Smagghe G (2014). CCK(-like) and receptors: Structure and phylogeny in a comparative perspective. Gener. Comp. Endocrinol..

[13] Nässel DR, Wu SF (2022). Cholecystokinin/sulfakinin peptide signaling: conserved roles at the intersection between feeding, mating and aggression. Cell Mol. Life Sci..

[14] Tinoco, A. B. *et al.* Ancient role of sulfakinin/cholecystokinin-type signalling in inhibitory regulation of feeding processes revealed in an echinoderm. *eLife* **10**, e65667, 10.7554/eLife.65667 (2021). 10.7554/eLife.65667 PMC8428848 34488941

[15] Nachman RJ, Holman GM, Haddon WF, Ling N (1986). Leucosulfakinin, a sulfated insect neuropeptide with homology to gastrin and cholecystokinin. Science.

[16] Nichols R, Schneuwly SA, Dixon JE (1988). Identification and characterization of a Drosophila homologue to the vertebrate neuropeptide cholecystokinin. J. Biol. Chem..

[17] Johnsen AH, Rehfeld J (1990). Cionin: a disulfotyrosyl hybrid of cholecystokinin and gastrin from the neural ganglion of the protochordate *Ciona intestinalis*. J. Biol. Chem..

[18] Sekiguchi T, Ogasawara M, Satake H (2012). Molecular and functional characterization of cionin receptors in the ascidian, *Ciona intestinalis*: the evolutionary origin of the vertebrate cholecystokinin/gastrin family. J. Endocrinol..

[19] Sekiguchi T, Kawashima T, Satou Y, Satoh N (2007). Further EST analysis of endocrine genes that are preferentially expressed in the neural complex of *Ciona intestinalis*: receptor and enzyme genes associated with endocrine system in the neural complex. Gen. Comp. Endocrinol..

[20] Chiba S, Sasaki A, Nakayama A, Takamura K, Satoh N (2004). Development of *Ciona intestinalis* juveniles (through 2nd ascidian stage). Zool. Sci..

[21] Delsuc F, Brinkmann H, Chourrout D, Philippe H (2006). Tunicates and not cephalochordates are the closest living relatives of vertebrates. Nature.

[22] Gandhi S, Razy-Krajka F, Christiaen L, Stolfi A (2018). CRISPR Knockouts in *Ciona* Embryos. Adv. Exp. Med. Biol..

[23] Sasakura Y, Horie T (2023). Improved genome editing in the ascidian *Ciona* with CRISPR/Cas9 and TALEN. Methods Mol. Biol..

[24] Sasakura Y, Yoshida K, Treen N (2017). Genome editing of the ascidian *Ciona intestinalis* with TALE nuclease. Methods Mol. Biol..

[25] Kawada T, Sekiguchi T, Sakai T, Aoyama M, Satake H (2010). Neuropeptides, hormone peptides, and their receptors in *Ciona intestinalis*: an update. Zoolog. Sci..

[26] Satake, H. Kobayashi Award 2021: Neuropeptides, receptors, and follicle development in the ascidian, *Ciona intestinalis *Type A: New clues to the evolution of chordate neuropeptidergic systems from biological niches. *Gen. Comp. Endocrinol.*, 114262, 10.1016/j.ygcen.2023.114262 (2023). 10.1016/j.ygcen.2023.114262 36925021

[27] Satake H (2019). Neuropeptides, peptide hormones, and their receptors of a tunicate. Ciona intestinalis. Results Probl. Cell Differ..

[28] Shiraishi A (2019). Repertoires of G protein-coupled receptors for *Ciona*-specific neuropeptides. Proc. Natl. Acad. Sci. USA.

[29] Osugi T, Miyasaka N, Shiraishi A, Matsubara S, Satake H (2021). Cionin, a vertebrate cholecystokinin/gastrin homolog, induces ovulation in the ascidian *Ciona intestinalis* type A. Sci. Rep..

[30] Petkova-Kirova P, Giovannini MG, Kalfin R, Rakovska A (2012). Modulation of acetylcholine release by cholecystokinin in striatum – receptor specificity; role of dopaminergic neuronal activity. Brain Res. Bull..

[31] Phillips PA (2010). Pancreatic stellate cells produce acetylcholine and may play a role in pancreatic exocrine secretion. Proc. Natl. Acad. Sci. USA.

[32] Hozumi A, Horie T, Sasakura Y (2015). Neuronal map reveals the highly regionalized pattern of the juvenile central nervous system of the ascidian *Ciona intestinalis*. Dev. Dyn..

[33] Florey E (1967). Cholinergic neurons in tunicates: An appraisal of the evidence. Comp. Biochem. Physiol..

[34] Kuzmin V, Volkova E, Sukhova G (2012). Cholinergic regulation of body-wall muscle contraction of the ascidian Styela rustica (Linnaeus, 1767). Russian J. Marine Biol..

[35] Jokura, K., Nishino, J. M., Ogasawara, M. & Nishino, A. An α7-related nicotinic acetylcholine receptor mediates the ciliary arrest response in pharyngeal gill slits of *Ciona*. *J. Exp. Biol.* **223**, 10.1242/jeb.209320 (2020). 10.1242/jeb.209320 32220975

[36] Ogasawara M (2002). Gene expression profiles in young adult *Ciona intestinalis*. Dev. Genes Evolut..

[37] Takamura, K., Egawa, T., Ohnishi, S., Okada, T. & Fukuoka, T. Developmental expression of ascidian neurotransmitter synthesis genes. I. Choline acetyltransferase and acetylcholine transporter genes. *Dev. Genes Evol.* **212**, 50–53, 10.1007/s00427-001-0205-0 (2002). 10.1007/s00427-001-0205-0 11875658

[38] Ikuta T, Saiga H (2007). Dynamic change in the expression of developmental genes in the ascidian central nervous system: revisit to the tripartite model and the origin of the midbrain-hindbrain boundary region. Dev. Biol..

